# Detection of Appearing and Disappearing Objects in Complex Acoustic Scenes

**DOI:** 10.1371/journal.pone.0046167

**Published:** 2012-09-27

**Authors:** Francisco Cervantes Constantino, Leyla Pinggera, Supathum Paranamana, Makio Kashino, Maria Chait

**Affiliations:** 1 Ear Institute, University College London, London, United Kingdom; 2 NTT Communication Science Laboratories, NTT Corporation, Atsugi, Japan; Baycrest Hospital, Canada

## Abstract

The ability to detect sudden changes in the environment is critical for survival. Hearing is hypothesized to play a major role in this process by serving as an “early warning device,” rapidly directing attention to new events. Here, we investigate listeners' sensitivity to changes in complex acoustic scenes—what makes certain events “pop-out” and grab attention while others remain unnoticed? We use artificial “scenes” populated by multiple pure-tone components, each with a unique frequency and amplitude modulation rate. Importantly, these scenes lack semantic attributes, which may have confounded previous studies, thus allowing us to probe low-level processes involved in auditory change perception. Our results reveal a striking difference between “appear” and “disappear” events. Listeners are remarkably tuned to object appearance: change detection and identification performance are at ceiling; response times are short, with little effect of scene-size, suggesting a pop-out process. In contrast, listeners have difficulty detecting disappearing objects, even in small scenes: performance rapidly deteriorates with growing scene-size; response times are slow, and even when change is detected, the changed component is rarely successfully identified. We also measured change detection performance when a noise or silent gap was inserted at the time of change or when the scene was interrupted by a distractor that occurred at the time of change but did not mask any scene elements. Gaps adversely affected the processing of item appearance but not disappearance. However, distractors reduced both appearance and disappearance detection. Together, our results suggest a role for neural adaptation and sensitivity to transients in the process of auditory change detection, similar to what has been demonstrated for visual change detection. Importantly, listeners consistently performed better for item addition (relative to deletion) across all scene interruptions used, suggesting a robust perceptual representation of item appearance.

## Introduction

The ability to detect and quickly respond to new events in the environment is critical to an organism's struggle for survival. The issue of sensitivity to change has been a topic of intense investigation in vision [1], [2]. Accumulating evidence has demonstrated that the visual system is highly sensitive to local transients (rapid changes in luminance/colour in a small section of the retinal image) such as would be generated by the abrupt appearance, disappearance or movement of objects within the scene. These events automatically draw attention towards the locations where they occur [3] resulting in perceptual ‘pop-out’ of the changing element. Conversely, when local, change-related, transients are masked, e.g. by a global transient (experiments commonly use a blank or random-noise screen presented between the original and changed images) subjects demonstrate a profound loss of sensitivity to change - an effect which has been termed ‘change blindness’ [1], [2], [4]–[6].

In contrast, the factors that affect listeners' ability to detect the appearance or disappearance of objects within busy acoustic scenes comprised of multiple concurrent sources remain poorly understood. This is despite the fact that sound is often what alerts us to important changes around us: Hearing is sensitive to a much wider space than the other senses and in many cases we *hear* a change before we see it (E.g. somebody walking into the room when our back is to the door). Indeed, the auditory system is commonly assumed to play a key role in the brain's change-detection network by serving as an ‘early warning device’, rapidly directing attention to new events in the scene [7], [8].

We are aware of only a handful of studies that examined listeners' sensitivity to change in scene contents [9]–[12]. All used ‘scenes’ comprised of concurrently presented naturalistic sounds (animal/human vocalizations, environmental sounds, etc.) and participants were instructed to detect salient changes - appearance, disappearance or switch in the location of an object. The results revealed a general difficulty with auditory change detection, referred to as ‘*change deafness*’, which occurred for even very small scenes (4 objects). Pavani and Turatto [10] further demonstrated no difference between conditions where the pre- and post-change scenes were contiguous or separated by silent or noise-filled gaps. The explanation offered to this set of findings is that rather than low-level sensory mechanisms, auditory change detection fundamentally relies on limited-capacity acoustic memory [10] and is not automatic, in that it requires directed attention [11], suggesting, rather surprisingly, that the auditory system may not be as sensitive to change as previously assumed.

However, this interpretation is confounded by the use of easily identifiable natural sounds. The use of natural sounds in the laboratory poses several problems. First, it is hard to control their physical parameters (indeed they were not controlled in previous work), and thus difficult to relate specific effects to underlying stimulus properties. For example, because the previously used sounds overlapped in frequency, inter-element masking grew as scenes became more populated and this might have contributed to the observed deterioration of performance with growing scene size. Second, the sounds were familiar and associated with semantic labels (in many cases subjects were explicitly encouraged and trained to name the sounds, e.g. ‘dog’, ‘cello’). It is thus difficult to exclude the possibility that instead of detecting ‘change in sound’ per se, listeners employed a strategy of explicitly scanning and memorizing the labels of the sources present in the beginning of the stimulus and comparing those to the ones present towards its end. The performance limits may consequently reflect limits of general working memory rather than a specific auditory change detection system (see also [13]).

**Figure 1:**
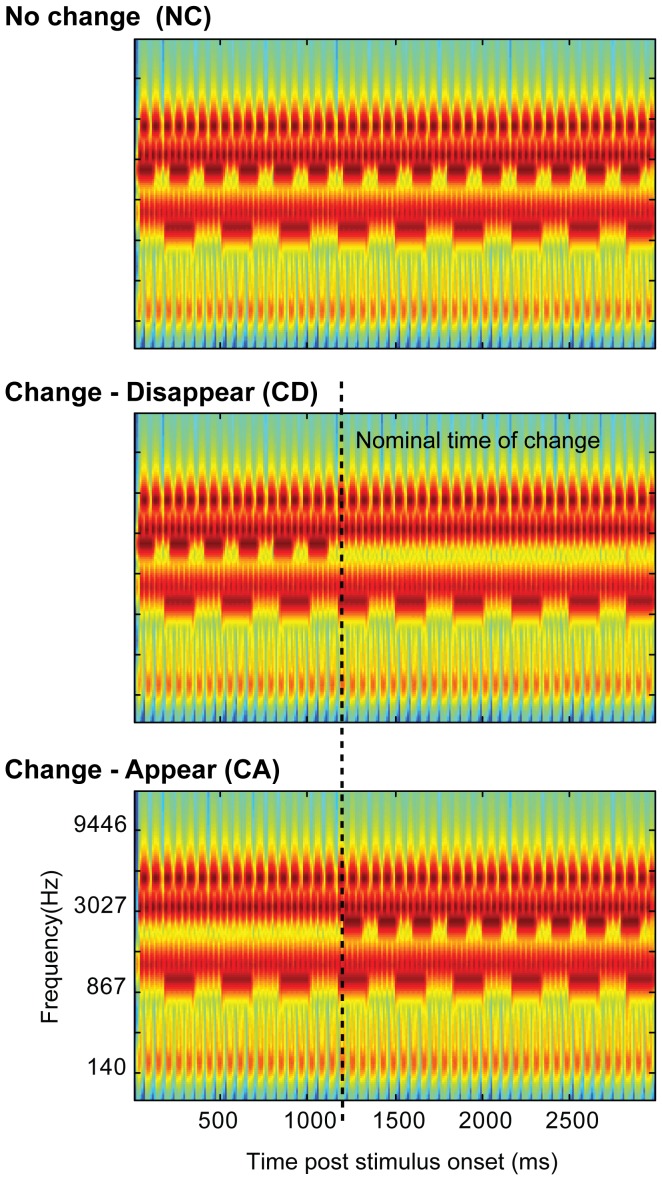
Example of the experimental stimuli. A: ‘no-change’ (NC) stimulus with six components. B and C show the ‘change-disappear’ (CD) and ‘change-appear’ (CA) variations. Dashed lines show the nominal change time. The plots represent ‘auditory’ spectrograms, generated with a filterbank of 1/ERB wide channels [14] equally spaced on a scale of ERB-rate. Channels are smoothed to obtain a temporal resolution similar to the Equivalent Rectangular Duration [41].

Here we developed a new paradigm for studying auditory change processing. We use artificial ‘scenes’ (Figure 1) populated by multiple streams of pure-tones designed to model acoustic sources. Each source is characterized by a different frequency, and is furthermore modulated at a distinct amplitude modulation (AM) rate to ensure that sources are perceived as separate, distinguishable items. The modulation mimics temporal properties found across many natural sounds (e.g. an engine's hum or a bird's chirp).

In a series of psychophysical experiments we measured listeners' ability to detect changes (in the form of appearance or disappearance of objects) in such ‘soundscapes’. To do so, listeners must be able to identify specific onset/offset events associated with the addition or deletion of scene elements out of the multitudes of onset and offset transients characterizing the on-going components. Scenes were presented continuously or with various interruptions, with the aim of understanding what makes certain changes ‘pop-out’ and grab attention while others remain un-noticed.

## General Methods

### Ethics Statement

Experimental procedures were approved by the research ethics committee of University College London, and written informed consent was obtained from each participant. Subjects were paid for their participation.

### Subjects

About 10 paid subjects (specific information detailed below) took part in each experiment (each experiment consisted of a different group of subjects but many participated in more than one). All reported normal hearing and no history of neurological disorder. The vast majority were not musically trained.

### Stimuli

Figure 1 presents an example of the experimental ‘acoustic scene’ stimuli. Scenes comprised multiple components, each consisting of a pure tone modulated by a square wave. Square wave steps were shaped by 3 ms raised-cosine ramps. Each component had a unique frequency and AM rate. Component frequencies were randomly drawn from a pool of 15 fixed values between 100 and 5000 Hz spaced at 2*ERB [Equivalent rectangular bandwidth; 14]. Component AM rates were randomly drawn from a pool of 15 fixed values between 3 and 35 Hz (random phase). Unless otherwise specified, scenes consisted of 4, 8, or 14 components. Stimulus duration varied randomly between 2000 and 4000 ms (in steps of 100 ms). We refer to scenes in which each source is active throughout the stimulus, as ‘no change’ stimuli (NC). Additionally, versions in which a single component is removed partway through the scene (‘change-disappear’, CD, stimuli), and versions in which the same single component is added to the scene (‘change-appear’, CA, stimuli) were created (see Figure 1B,C). The timing of change varied randomly between 1000 and 2000 ms post scene onset with the constraint that changed components were added or deleted with zero phase: for appearing components, the nominal time of change was set at the introduction of the first non-zero sound sample to the scene; for disappearing components the time of change was the time at which the next tone-burst was expected to appear (see dashed lines in Figure 1B,C). The choice of component frequencies and AM rates was random for each scene, but to enable a controlled comparison between CA and CD signals, the experimental stimuli were generated as NC/CD/CA triplets (as in Figure 1), containing identical components. These were then presented in random order (or blocked according to change type) during the experiment. Thus NC, CD, and CA scenes had *overall* the same frequency and AM content.

Stimuli were constructed in MATLAB (The Mathworks, USA) at a sampling rate of 44100 Hz with 30 ms raised cosine ramps and presented with an EDIROL UA- 4FX sound card (Roland Corporation) over high quality headphones (Sennheiser HD 555) at a comfortable listening level (∼60–70 dB SPL), self-adjusted by each participant. Stimulus presentation was controlled using the *Cogent* software (http://www.vislab.ucl.ac.uk/cogent.php). Stimulus conditions specific to each experiment are described below.

### Procedure

The experiment was conducted in an acoustically-sealed booth (IAC, Winchester, UK). Experimental sessions lasted between 1.5 to 2 hours and consisted of a short practice session with feedback, followed by the main experiment with no feedback, divided into runs of about 10 minutes. Subjects were instructed to fixate at a cross presented on the computer screen, while performing the task relevant to each experiment. Participants were allowed a short rest between runs.

### Analysis

Dependent measures are hit rate, *d*′ score [15] (where applicable) and response time (RT; measured between the nominal time of change and the subject's key press). Where available, the statistical analysis is based on the *d′* score as it provides a more accurate measure of sensitivity than hit rate. When hit rate = 100% or false positive rate = 0 (resulting in undefined *d*′) these were adjusted to 99.9999 and 0.0001, leading to a maximum obtainable *d*′ of 8.6. The α level was *a priori* set to 0.05.

## Experiment 1

The purpose of Experiment 1 was to determine whether our artificial scenes are suitable for assessing change detection. We aimed to measure: a) the extent to which listeners are able to selectively attend to a single component within a scene, and b) their ability to retrospectively determine whether a given component had been present within a just-heard scene.

### Subjects

Ten subjects participated in the experiment (8 female; mean age = 25.2 years).

### Stimuli and Methods

Experiment 1 used only NC stimuli, associated with a probe in two configurations (presented in separate blocks): ‘probe - NC’ (probe followed by scene) and ‘NC - probe’ (scene followed by probe). The probe consisted of a single component (amplitude-modulated tone). The duration of the scene was 2000 ms, that of the probe 1000 ms, and NC and probe were separated by a 200 ms silence. The probe component was either present or absent within the NC scene, with a probability of 0.5. Subjects had to determine whether the component was present. Probe-scene pairs were presented with an inter-trial-interval randomized between 900 and 2200 ms.

### Results

**Figure 2:**
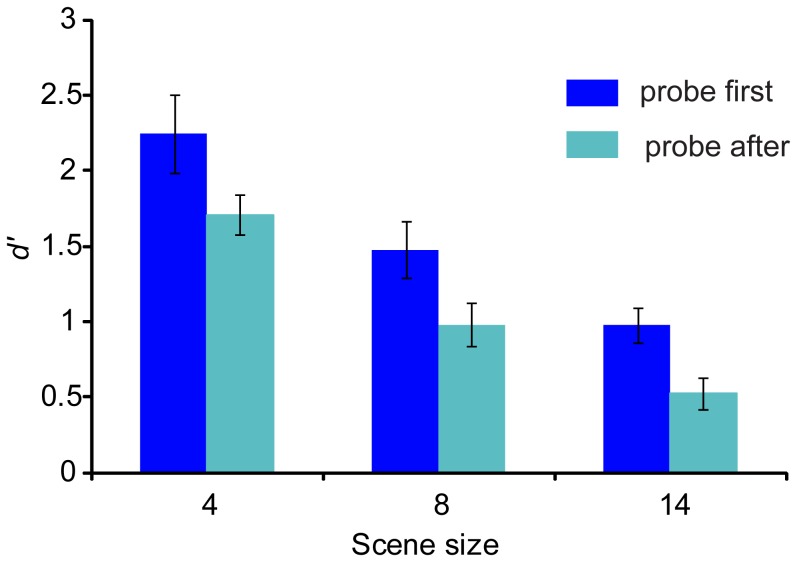
Results of **Experiment 1** **which tested listener's ability to judge whether a probe (a single AM tone) is present within a NC scene.** Plotted are sensitivity scores as a function of scene size. Dark blue: probe presented before the scene; light blue: probe presented after the scene. Error bars are 1 standard error (SE).

Figure 2 plots the results of Experiment 1. As expected, performance is better for smaller scenes, and furthermore better if the probe precedes the scene. A repeated measures ANOVA on *d′* scores with task (probe-NC, versus NC-probe), and scene size as factors, showed a main effect of task (F(1,9) = 17.473; *p = 0.002*), and a main effect of scene size (F(2,18) = 73.59; *p<0.001*) with no interaction. Performance was above floor (*d′*>0.5) for all ‘probe - NC’ conditions, and for the 4- and 8-component scenes in the ‘NC - probe’ condition.

Reasonable performance on ‘probe-NC’ suggests that listeners are able to selectively segregate and hear out individual components. The data also demonstrate that listeners are able, to a certain extent, to determine, *post hoc* whether a particular component was part of the scene.

These results demonstrate that, similarly to natural scenes, the artificial scene stimuli used here, are perceived as a ‘sound-scape’ that is perceptually separable (as opposed to a single fused sound) and are therefore suitable for the study of change detection.

## Experiment 2

The goal of Experiment 2 was to test listeners' ability to detect sudden changes in the scene, manifested as the appearance or disappearance of an element.

### Subjects

Seven subjects participated in the experiment (4 female; mean age = 25.3 years).

### Stimuli and Methods

We used a broad range of scene sizes (4, 6, 8, 10, 12, and 14 components). All stimuli were presented in random order (not blocked according to change type) with an inter-stimulus-interval (ISI) between 700 to 2000 ms. To maintain an equal proportion (50%) of change and no-change stimuli, additional NC stimuli (without matching CA and CD versions) were added to the stimulus set. Subjects were required to press a button once they detected a change in the scene (they were not required to determine the type of change).

### Results

**Figure 3:**
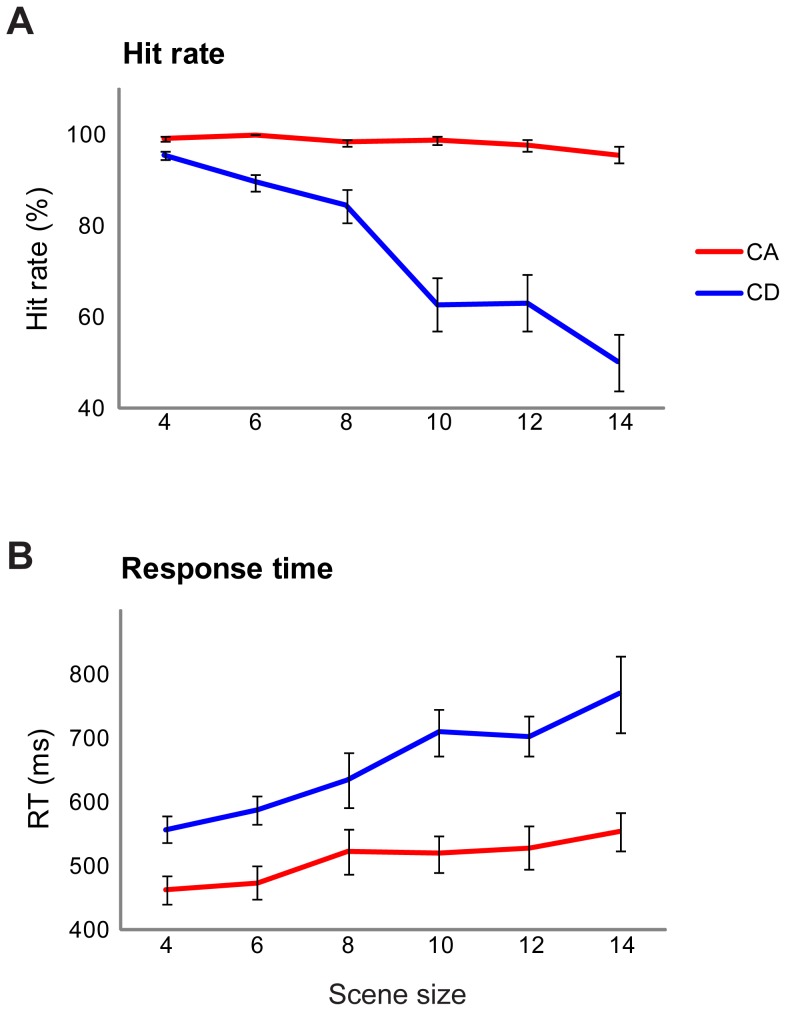
Results of the main change detection task ( **Experiment 2** **). Error bars are 1 SE.**

Results are shown in Figure 3. There is a remarkable difference in performance for CA versus CD stimuli: while subjects remained at ceiling performance for CA stimuli for all scene sizes, there was a sharp decrease in CD hit rates as scenes became more populated (Figure 3A). A repeated measures ANOVA on hit rate data, with change type (CA versus CD) and scene size as factors, showed a main effect of change type (F(1,6) = 47.87; *p<0.001*), a main effect of scene size (F(5,30) = 36.42; *p<0.001*), and an interaction (F(5,30) = 24.168; *p<0.001*). To examine the interaction, repeated measures ANOVA revealed no effect of scene size for CA (F(5,30) = 2.81; *p = 0.101*), but a strong effect (F(5,30) = 31.92; *p<0.001*) for CD stimuli. The interaction suggests that performance decreased with scene size in CD trials, whereas scene size had no effect on CA stimuli. For reaction times (Figure 3B) a similar analysis showed a main effect of change type (F(1,6) = 166.48; p<0.001), a main effect of scene size (F(1.565,9.392) = 23.48; p<0.001) and an interaction (F(2.25,13.498) = 4.55; p = 0.028). To examine the interaction, repeated measures ANOVA demonstrated an effect of scene size for both CA (F(1.994,11.964) = 11.03; *p = 0.002*) and CD (F(1.836,11.014) = 14.88; *p* = 0.001) stimuli, with the observed interaction indicating a sharper slope in the case of CD trials. Overall, listeners performed substantially better and faster on CA relative to CD stimuli. *d*′ scores could not be calculated due to the randomly interleaved presentation of CD and CA scenes. However, Experiment 5 (below) replicated the same pattern of results in a blocked design for which the computation of *d*′ was possible.

## Experiment 3

The addition of a component (in a CA scene) is associated with a slight loudness increase, while the deletion of a component (in a CD scene) results in a slight loudness decrease. It could well be that subjects were solving the change detection task by using *loudness change* as a cue. Indeed, it has been established that listeners are more sensitive to loudness increments than decrements (e.g. [16], [17]), which might explain the behavioural asymmetry in CA versus CD. In the following experiment we evaluated the extent to which sensitivity to loudness change may have played a role in listeners' performance. Specifically, we introduced prominent (at least 6 dB) loudness changes to all signals (CA, CD, and NC; at the nominal time of change), which were large enough to mask any loudness-related cues involved in item addition or deletion (estimated to be at most 6 dB, in scenes with 4 components, and lower for larger scenes). We reasoned that if the performance in Experiment 2 is mainly due to sensitivity to loudness, then the effect of change type (the CA/CD disparity) should vanish when these cues are systematically masked.

### Subjects

Ten subjects (5 female; mean age = 23.6 years) participated in Experiment 3A. Five subject (4 female; mean age = 25.4 years) participated in Experiment 3B.

### Stimuli and Methods

Stimuli in Experiment 3a were identical to those in Experiment 2 except that a loudness change in the form of a single upwards or downwards step in amplitude was introduced in all stimuli, at the nominal change time. The magnitude and direction of the loudness change were chosen randomly from a pool of 6 steps (−18, −12, −6, 6, 12, or 18 dB). We used a variety of step sizes in order to make the magnitude as well as the direction of loudness change (increase vs. decrease) unpredictable. This would encourage the listeners to ignore the loudness change and focus on the change in scene content. If each component contributes equally to the loudness of the overall scene, disappearance/appearance of an element in a 4 component scene would result in a power change of 1.25 dB and smaller for larger scenes. However the loudness of some components could be perceived as larger because of unequal sensitivity to frequency. Setting the smallest step size at 6 dB is a conservative estimate designed to address this eventuality.

As in Experiment 2, stimuli were generated in NC/CA/CD triplets, such that NC scenes also contained a step-change in loudness at the same time as their matching CA and CD stimuli. Subjects were instructed to ignore the loudness changes and focus on detecting a change in the content of the scene.

Experiment 3b used the same stimuli, except that different change types (CA, CD) were blocked separately to allow the computation of a *d′* score.

### Results

**Figure 4:**
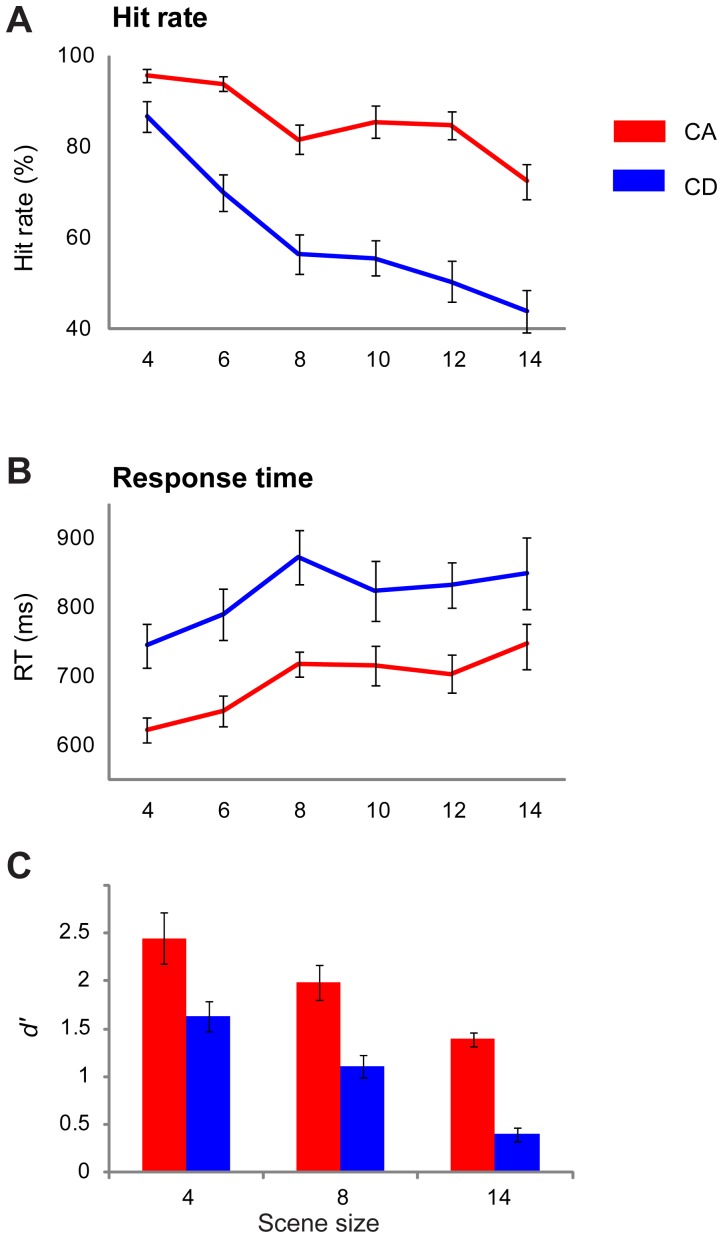
Results of **Experiment 3** **.** A loudness change in the form of a single upwards or downwards step in amplitude was introduced in all stimuli, at the nominal change time. A, B show results from Experiment 3A (randomized presentation). C shows d′ data obtained in Experiment 3B where the same stimuli were presented in blocks according to change type (CA or CD). Error bars are 1 SE.

Figure 4 presents the hit rate (Figure 4A) and RT (Figure 4B) data. Performance in this experiment, for both CA and CD, is generally worse than in Experiment 2. This is expected because the addition of the loudness change, which subjects are required to learn to ignore, makes the change detection task harder. Nevertheless the pattern of results is similar to that of Experiment 2. Importantly, the differential performance on CA vs. CD is maintained.

A repeated measures ANOVA on hit rate data, with change type (CA versus CD) and scene size as factors, showed a main effect of change type (F(1,9) = 85.56; *p<0.001*), and a main effect of scene size (F(1.93,17.4) = 46.17; *p<0.001*) with an interaction (F(2.57,23.13) = 7.84; *p = 0.001*). To examine the interaction, repeated measures ANOVA revealed an effect of scene size for both CA (F(5,45) = 17.72; *p<0.001*), and CD (F(1.96,17.64) = 35.96; *p<0.001*). The interaction suggests that the scene size effect is stronger for CD than CA trials. For RT, the same analysis showed a main effect of change type (F(1,9) = 37.89; *p<0.001*) and a main effect of scene size (F(5,45) = 9.56; *p<0.001*) with no interaction. Overall, the statistical tests therefore confirm that listeners performed significantly better and faster in detecting appearing rather than disappearing components. This suggests that the asymmetry between CA and CD is ***not*** based on sensitivity to loudness change cues.

In **Experiment 3** **B** we ran a blocked version of this study in order to obtain *d′* scores. Those results similarly demonstrated preserved CA/CD asymmetry (Figure 4C).

## Experiment 4

In Experiment 4 we assessed participants' ability to identify the appearing or disappearing components. That is, we sought to measure whether, in addition to detecting that some change has occurred, listeners have access to more detailed information about *what* changed. To do this, a secondary probe recognition task was administered during trials in which a change was detected.

### Subjects

Ten subjects participated in Experiment 4A (5 female; mean age = 24.8 years). Ten additional subjects (3 female; mean age = 25.9) participated in Experiment 4B. Two of the participants were musically trained.

### Stimuli and Methods

The stimuli in Experiment 4A were identical to those in Experiment 2 except that each time the participant pressed a button to indicate that change was detected, the scene was interrupted and a probe (1000 ms amplitude modulated pure tone) was presented 200 ms later. The probe was either the component associated with the change or one of the other components present in the scene, with equal probability. Subjects were required to judge whether the probe was identical to the changed component. They were encouraged to focus primarily on the change detection task, and guess if unsure about the probe task. So as to not inadvertently provide feedback, probes were also presented after false positives (response to a NC stimulus), but those trials were not analysed. Experiment 4B was identical except that the probe carrier frequency was fixed at 500 Hz and subjects were required to respond if its AM rate was identical to that of the changed component.

### Results

The results of the change detection task (not shown) replicated those of Experiment 2.

**Figure 5:**
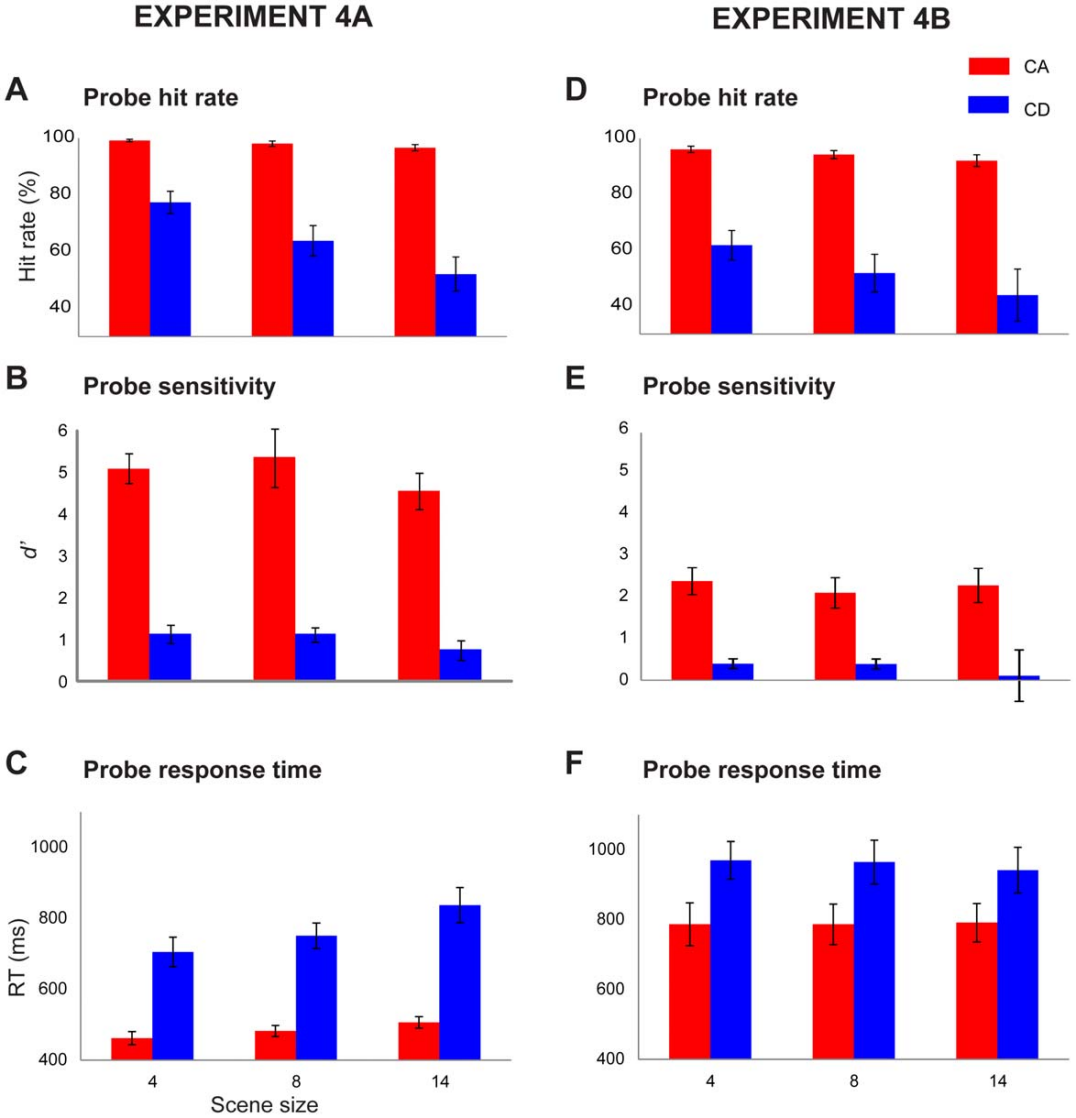
Results of Experiment 4 which tested listeners' ability to identify the component that appeared or disappeared in CA and CD scenes. In Experiment 4A (left) the probe was identical to the changed component (frequency+AM). In Experiment 4B (right) the probe carrier frequency was fixed at 500 Hz and only the AM varied. Error bars are 1 SE.

The results from the probe task in **Experiment 4** **A** are presented in Figure 5A, B, C: A repeated measured ANOVA on *d′* scores, with change type and scene size as factors, showed a strong main effect of change type (F(1,9) = 372.70; *p<0.001*) but no effect of scene size (F(2,18) = 1.54; *p = 0.246*), with no interaction (F(2,18) = 0.16; *p = 0.851*). Listeners were apparently at ceiling for identifying the component that changed within a CA stimulus, but performed poorly with CD stimuli irrespective of scene size.

A repeated measures ANOVA on probe reaction times (Figure 5C), with change type and scene size as factors, showed a main effect of change type (F(1,9) = 72.09; *p<0.001*) and a main effect of scene size (F(1,18) = 12.73; *p<0.001*) with a weak interaction (F(2,18) = 3.85; *p = 0.04*). To examine the interaction, repeated measures ANOVA revealed an effect of scene size for CA stimuli (F(2,18) = 9.36; *p = 0.002*), and for CD (F(2,18) = 8.61; *p = 0.004*).

As a further test of change identification ability, in **Experiment 4** **B** the frequency of the probe was fixed at 500 Hz and listeners were required to respond when its AM was identical to that of the changed component. These results are presented in Figure 5D,E,F. As expected, this version of the task was generally more difficult because it required listeners to specifically remember the AM rate of the changing component. However participants still exhibited a striking difference between CA and CD changes. A repeated measured ANOVA on *d′* scores, with change type and scene size as factors, showed a main effect of change type (F(1,9) = 27.39; *p = 0.001*) but no effect of scene size (F(2,16) = 0.2; *p = 0.740*). Listeners are thus fairly accurate at identifying appearing components, irrespective of scene size, but are essentially unable to identify the disappearing components. Note that, in all cases, probes were presented only after trials in which the change was successfully detected by the listener, suggesting that, even though they *heard* the change in the CD scene, they were largely impaired at determining which component changed.

To investigate the extent to which memory limitation may have underpinned the poor performance on CD stimuli (under the assumption that CD change detection involves comparing the content in the beginning and end of scene), we compared (independent sample t test) performance on scene size 4 in Experiment 1 (scene-probe condition), with that in Experiment 4A. The results demonstrate significantly better performance in the former (*d′* of 1.7 relative to 1.1;t = −2.28; df = 18 *p* = 0.035).

## Experiment 5

The ‘appearance’ advantage observed in the previous experiments could be accounted for in terms of neural adaptation: In a putative, tonotopically organized, coding array, the introduction of a new component results in a ‘local spectral peak’ (higher firing rate to the newly added component than to the previously present scene elements). Such an account predicts improved CA performance when changes occur later from scene onset because adaptation would progressively weaken responses to the on-going scene elements. The present experiment was designed to test this prediction by measuring change detection performance as a function of change latency relative to scene onset.

### Subjects

Ten subjects participated in the experiment (4 female; mean age = 25.7 years).

### Stimuli and Methods

The stimuli in Experiment 5 were similar to those in Experiment 2 except that we systematically manipulated the nominal time of change. Stimulus duration varied between 3800 and 4200 ms. In ‘Early’ trials change occurred between 800 and 1200 ms post onset. In ‘Late’ trials change occurred 2000 ms later – between 2800 and 3200 ms post onset. Stimulus presentation was blocked by change-type (CA/CD) and change time (early/late). As before, the proportion of change events was 50% in each block. Block order was randomized across listeners.

### Results

**Figure 6:**
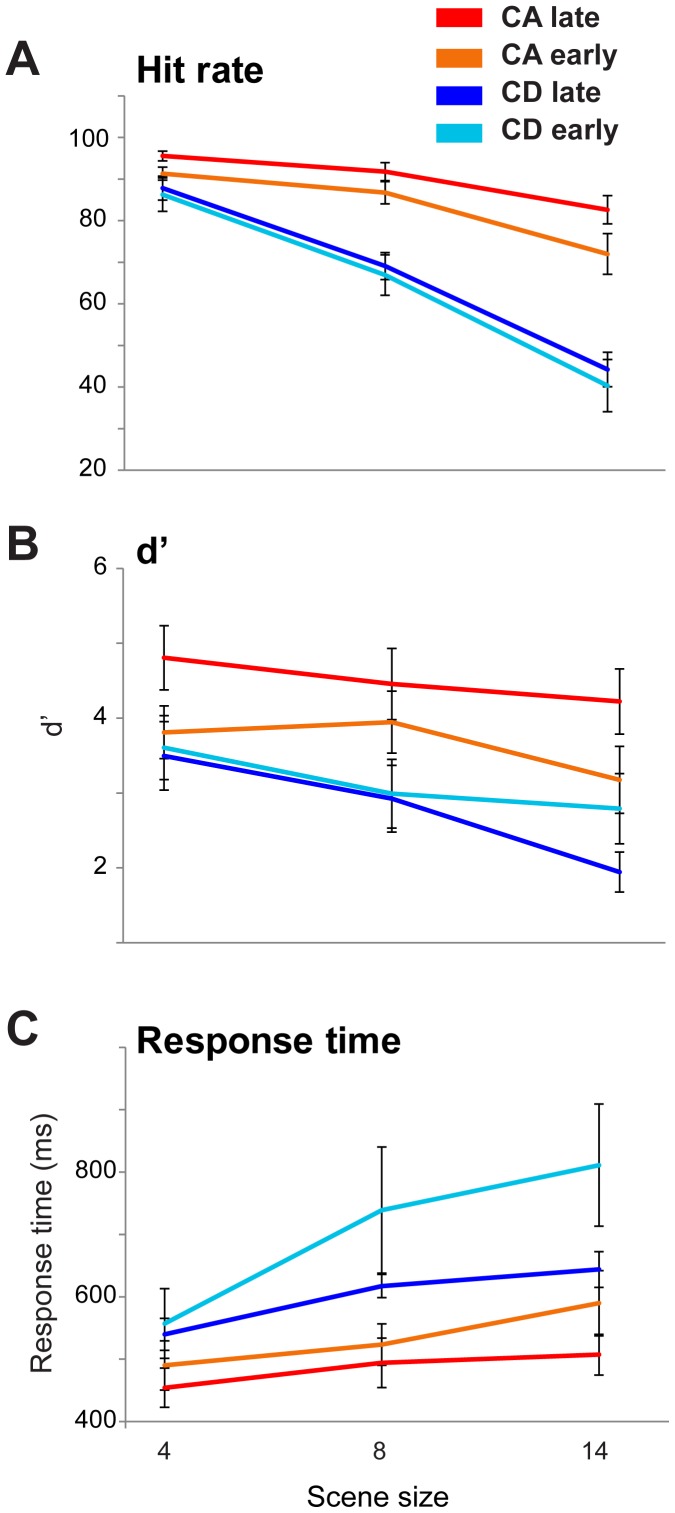
Results of Experiment 5, change detection as a function of time of change (early vs. late) for CA and CD. Error bars are 1 SE.

The results are presented in Figure 6A, B, C: A repeated measured ANOVA on *d′* scores, with change time, change type and scene size as factors, showed a main effect of change type (F(1,9) = 27.325; *p = 0.001*), scene size (F(2,18) = 12.93; *p<0.001*), and an interaction between change time and change type (F(1,9) = 9.62; p = 0.013). To examine the interaction, we conducted two separate repeated measures ANOVAs for CA and CD. These tests revealed a main effect of change time for CA (F(1,9) = 9.952; p = 0.012) but not for CD (F(1,9) = 0.77; p = 0.403). This pattern is also mirrored in the hit rate data. The results suggest therefore that shifting the time of change affects performance on CA - listeners are better at detecting appearance when it occurs later in the course of the scene. However, no such effect is observed for CD changes - performance does not improve for late, relative to early, changes. Despite the null effect on hit rate and *d′* data, response time data (Figure 6C) do show a substantial slowing down in the ‘CD early’ condition. However, the large variability of RT in that condition and the inconsistence with hit rate/sensitivity data make it hard to interpret.

Overall, the CA data are consistent with an account based on adaptation. It is also possible that the improvement in performance when changes occurred later in the scene stemmed from that, perceptually, listeners had more time to familiarize themselves with the on-going scene and thus new elements became easier to spot. In this context, the fact that CD performance did not improve in the ‘late’ condition, despite the fact that listeners had additional 2000 ms to scan the scene, is noteworthy and is consistent with the ‘disappearance deafness’ results from Experiment 4, above.

## Experiment 6

The previous experiment demonstrated that CA detection is supported by adaptation - progressive reduction of the sustained neuronal firing rate, in response to the signal while it is present in the scene. Another low level neuronal mechanism which might play a role in change detection is local transients. Appearance and disappearance of a component are associated with a ‘local transient’ - an abrupt change in stimulus power within a certain frequency band, resulting in a sharp change in the firing rate of a small number of neighbouring cells while activity in the rest of the array is unaltered. A mechanism which is sensitive to such transients might support change detection since it is able to indicate the time, the frequency region, and the nature of the change (appearance vs. disappearance).

It is difficult to discuss/probe ‘neural adaptation’ and ‘sensitivity to transients’ separately, because they likely work in concert to shape neural responses to change events. For simplicity, we refer to *adaptation* as the reduction in firing rate over time, and to *transients* as the actual onset/offset responses generated at the time of transition.

The sudden onset or offset of acoustic stimulation evokes robust neural responses across the auditory pathway within cells with receptive fields tuned to the stimulus frequency. These On- and Off- tuned cell populations are characterized by markedly different properties: It has recently been demonstrated that while many primary auditory cortical cells generated both On responses and Off responses, these are driven by separate sets of synapses, suggesting that the neural machinery for onset detection and offset detection are largely separate at that stage [18]. Furthermore, offset tuned cells are fewer in number, and their responses tend to be of longer latency and smaller amplitude [19]–[22], thus leading to larger, and earlier, on- than off- transient responses. These differences are consistent with our finding that appearance events are overall more detectable.

Computationally, detection of item addition in our stimuli should be relatively easy, as it is associated with appearance of energy within a frequency band that was previously inactive. Disappearance, on the other hand, is not easily distinguished from the many offsets that occur due to on-going modulation. Disappearance detection in the present stimuli requires a ‘smarter’, ‘second order transient’ detection mechanism, capable of acquiring the temporal patterning of the on-going sound, and signalling when those rules are violated, e.g. when an expected tone pip fails to arrive. The existence of such ‘smart’ offset detection mechanisms (albeit in the context of a single sequence rather than several concurrent sources), operating automatically irrespective of listeners' attentional focus, has been demonstrated in several recent human brain imaging studies [23]–[25] and it has been hypothesized that they might play a role in scene change detection. The animal electrophysiology literature has largely focused on offset responses to simpler sounds (long pure tones) however there is some evidence (e.g. [26]) of similar offset responses observable at the population level.

To assess the degree to which local energy-transients contribute to listeners change detection performance, we created stimuli in which they are masked by a global transient (a disruption that affects all frequency channels simultaneously) occurring at the nominal time of change, and measured the degree to which this disturbs performance.

Technically, the loudness change used in Experiment 3 constituted a general disruption which may have masked, at least to a certain extent, the transient associated with a component change. However, if the transient rides on this loudness change, it may still be detectable. Conceivably, the simplest form of global transient is a silence gap. This would cause a deactivation, or a *lull*, across the entire encoding array. At gap offset, all channels will be reactivated together, masking the transient specific to the added/deleted item. Alternatively, the transient can be masked by a gap that is filled with loud wide-band noise which is perceptually more distracting.

If change detection benefits from sensitivity to within-channel (local) transients, the global transient would abolish, or reduce, reliance on within-channel transients leading to reduction in performance. Under the hypothesis that local transients play a role in both CA and CD detection, we might expect that CD would be more severely affected by a global scene disruption because, as discussed above, Off-responses tend to be weaker in amplitude than On-responses.

### Subjects

Ten subjects participated in the experiment (5 female; mean age = 23.8 years).

### Stimuli and Methods

The stimulus set included three conditions: a) ‘no gap’ stimuli identical to the ones used in Experiment 2; b) ‘silent gap’ stimuli with a 200 ms silent gap inserted at the time of change; c) ‘noise gap’ stimuli with a 200 ms white noise burst inserted at the time of change. The noise level (18 dB above the level of the scene) was set to be just sufficient to mask the scenes. Gap duration (200 ms) was chosen to be as short as possible so as to minimise reliance on memory capacities, but longer than the longest inter-pulse interval (corresponding to the slowest AM rate used) in order to introduce a detectable gap for all scene components. The signals before and after the gap were ramped with a 10 ms cosine-squared ramp. For each gap condition, signals were generated as NC/CD/CA triplets such that NC signals also contained a gap at the same time as their matching CA and CD scenes. Stimulus presentation was blocked by change-type and gap-type (no-gap/silence/noise). As before, the proportion of change events was 50% in each block. Block order was randomized across listeners.

### Results

**Figure 7:**
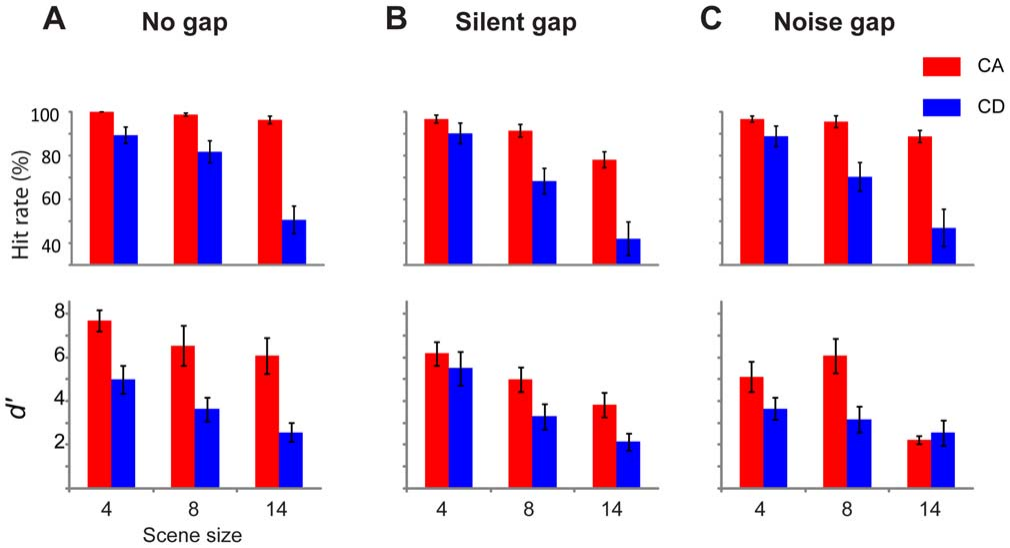
Results of Experiment 6, comparing performance on continuous scenes (A) and those where a silence (B) or noise-filled (C) gap was inserted at the time of change. Error bars are 1 SE.

**Figure 8:**
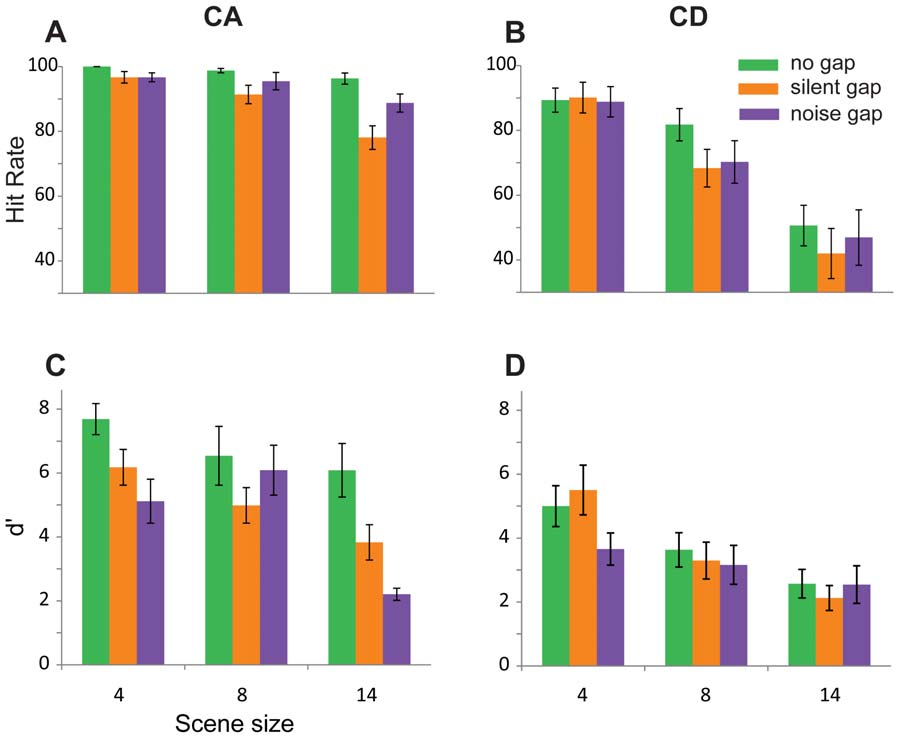
Results of Experiment 6 presented in a form that is relevant for interpreting the interaction of change type (CA vs. CD) and gap (continuous, vs. silence gap, vs. noise gap). The introduction of gaps adversely affected performance on CA but not CD changes. Error bars are 1 SE.

Figure 7 shows the results per gap type condition. A repeated measures ANOVA on *d′* data with gap type (continuous vs. silent vs. noise), change type (CA versus CD), and scene size as factors, showed main effects of gap type (F(2,18) = 18.85; *p<0.001*), change type (F(1,9) = 46.4; *p<0.001*), and scene size (F(2,18) = 28.55; *p<0.001*), with an interaction between gap and change type (F(2,18) = 14.33; *p<0.001*). The statistical test therefore confirms that listeners perform significantly better for CA than CD stimuli, across all gap conditions. Figure 8 presents the same data in a form relevant for interpreting the interaction. For CA stimuli: Repeated measures ANOVA reveals an effect of gap type (F(2,18) = 38.36; *p<0.001*), and of scene size (F(2,18) = 13.27; *p<0.001*), with an interaction (F(4,36) = 3.51; *p = 0.04*). This last interaction is due to continuous conditions showing no effect of scene size (F(2,18) = 1.46; *p = 0.257*) while the silence and noise gap conditions do (F(2,18) = 6.99; *p = 0.006 and F(2,18) = 12.68; p = 0.001*, respectively). To investigate the above main effect of gap type, a post hoc test (Benferroni adjusted for multiple comparisons) on the mean differences between continuous, noise, and silent-gap conditions revealed a significant difference between continuous and noise and continuous and silent-gap (both *p<0.001*) and no difference between noise and silent gap (p = 0.989). Thus, listeners are most sensitive to appearance of a component in a continuous scene, with silent and noise gaps being equally detrimental. The fact that gaps adversely affect CA pop-out is also suggested by the emergence of dependence on scene-size after gap introduction.

For CD stimuli: repeated measures ANOVA revealed no effect of gap type (F(2,18) = 1.66; *p* = 0.223), and a strong effect of scene size (F(2,18) = 30.94; *p*<0.001), with no interaction (F(4,36) = 1.12; *p* = 0.356). Thus, the introduction of gaps has no effect on CD stimuli, with sensitivity values remaining the same across all three gap conditions. Identical patterns are observed in the analysis of hit rate data. (Figure 7A,B).

The data suggest therefore that the introduction of a global transient had different effects on CA and CD changes: CA detection was significantly reduced while performance on CD remained intact, contrary to what was expected. Note that the CD null effect cannot be attributed to floor effects because baseline CD performance is well above floor.

It is possible that the results of Experiment 6 reflect the effects of adaptation rather than sensitivity to transients. The gaps were meant to serve as maskers for the change-related local transient but they might also have caused a re-setting of adaptation across the channels thereby reducing CA pop-out. Indeed the pattern of results observed here (reduction of performance on CA, no effect for CD) is similar to the pattern observed in Experiment 5, where we systematically manipulated the effects of adaptation. We have deliberately chosen very short gaps in order to minimize these effects (see methods) but cannot exclude the possibility that even a duration of 200 ms was sufficient to re-set adaptation.

## Experiment 7

**Figure 9:**
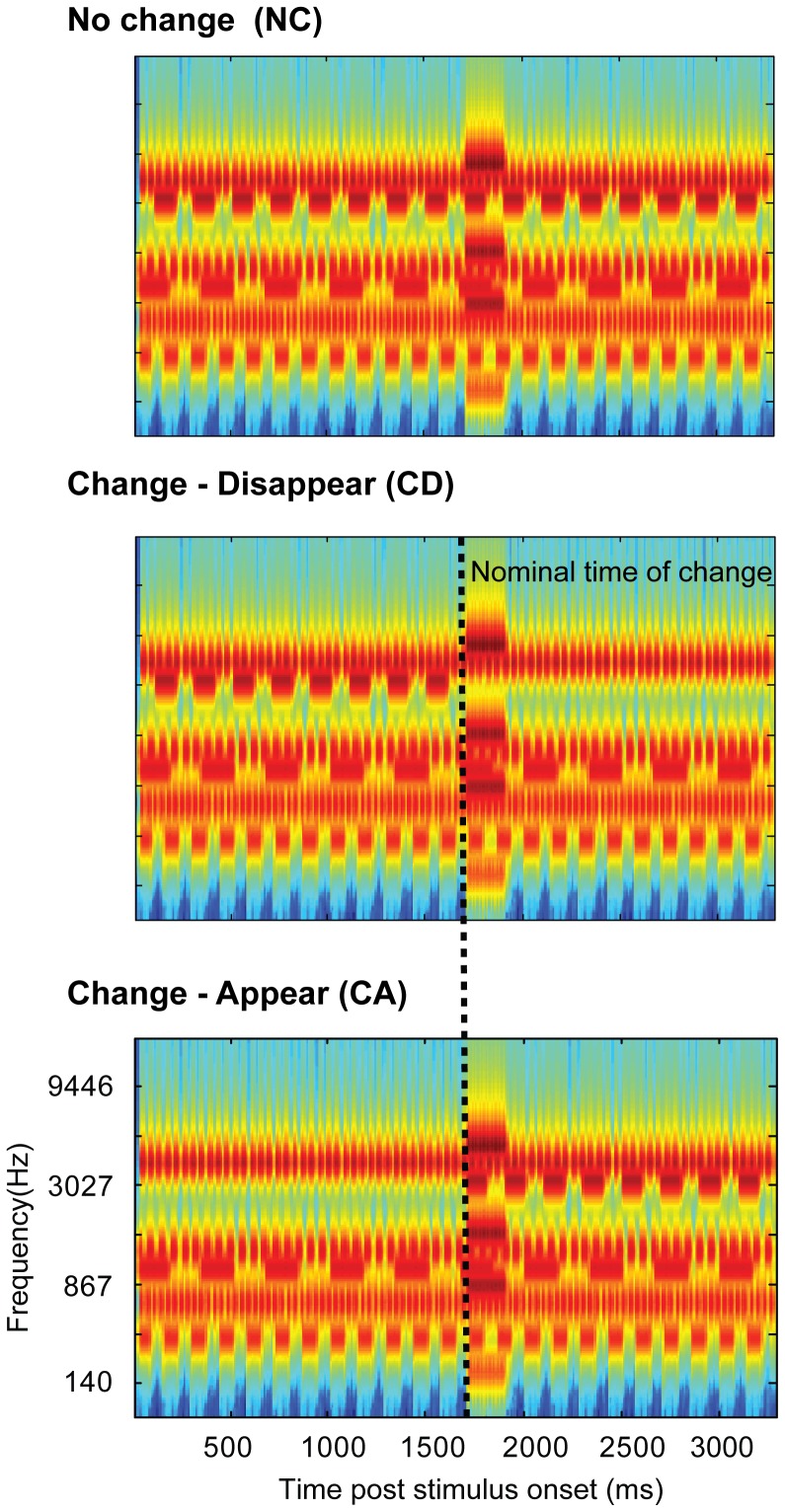
Example of the ‘splash’ stimuli. ‘Splashes’ are chords of four concurrently presented 200 ms tones, amplitude modulated at 100 Hz. They occur at the time of change but do not mask any scene components (‘Splash’ frequencies were chosen such that they were separated by at least 2 ERB from each scene component). A: ‘no-change’ (NC) stimulus with six components. B and C show the ‘change-disappear’ (CD) and ‘change-appear’ (CA) variations. Dashed lines show the nominal change time. The plots represent ‘auditory’ spectrograms, generated with a filter bank of 1/ERB wide channels [17] equally spaced on a scale of ERB-rate. Channels are smoothed to obtain a temporal resolution similar to the Equivalent Rectangular Duration [41].

In Experiment 7, we further investigate the possible role of sensitivity to change-related transients by using a different method for masking the onset/offset transients associated with item appearance or disappearance. Instead of introducing a global transient (as in Experiment 6), the scene stimuli in Experiment 7 include a brief interruption that occurs at the time of change but does not mask any scene components (Figure 9). Neural adaptation to the on-going scene elements is therefore not disrupted. The paradigm is inspired by the ‘mud splashes’ experiments of O'Regan et al [5], who demonstrated that visual change detection can be severely diminished by introducing brief, high-contrast, localized disruptions (‘mud splashes’) to an image. These ‘mud splashes’ do not physically obscure the location of the change, but rather ‘informationally’ mask the change-related transient by attracting attention away from the change location.

### Subjects

Twelve subjects participated in this experiment (9 female; mean age = 27.1 years).

### Stimuli and Methods

In Experiment 7 The stimulus set included two conditions: a) ‘non interrupted’ scenes identical to the ones used in Experiment 2; b) ‘splash’ scenes (nomenclature inspired by O'Regan et al, [5]) in which the scene was interrupted by an acoustic distractor (‘splash’; Figure 9). The ‘splashes’ are chords of four, concurrently presented, 200 ms pure tones. Tone frequencies were selected out of the remaining values from the frequency-bank used for the scene components (this strategy ensures that ‘splash’ components did not mask any scene elements) and thus varied from trial to trial. To make the ‘splash’ components ‘stand out’ as different from the scene elements, they were amplitude modulated at 100 Hz (depth of 0.5) and this sounded like a brief ‘buzz’ occurring partway through the scene. Scenes in this experiment were comprised of 4, 6, or 10 components. Note that this is different from the previous experiments and was necessary because ‘splash’ components and scene elements shared the same frequency pool. Similarly to the non-interrupted scenes, ‘splash’ scenes were created as NC/CA/CD triplets (as in Figure 9) and then presented in random order (blocked according to interruption type and change type) to the listeners.

### Results

**Figure 10:**
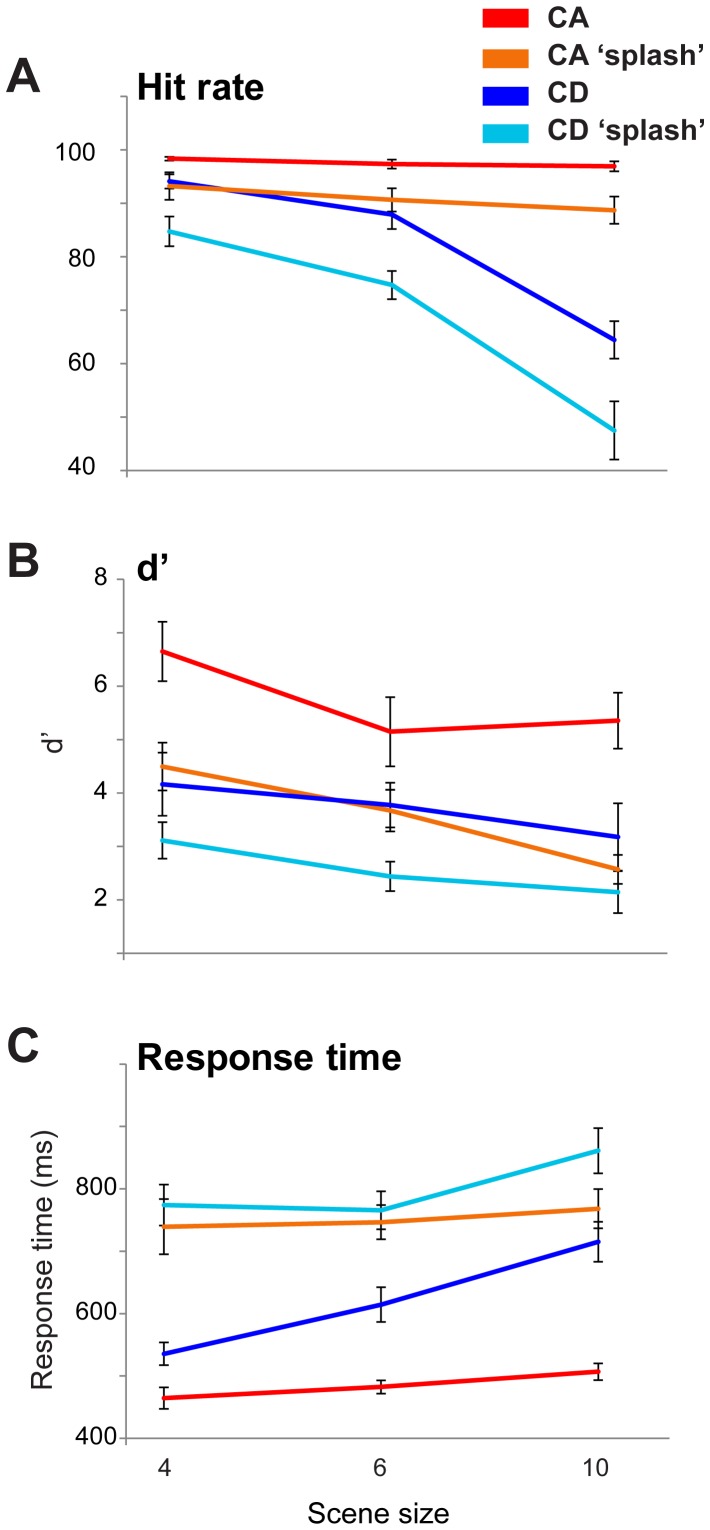
Results of Experiment 7, comparing performance on non-interrupted scenes and those where a brief acoustic ‘splash’ occurred at the time of change. Error bars are 1 SE. Note that the scenes sizes used here are different from the previous experiments. Reducing the maximum scene size to 10 (rather than 14) was necessary because ‘splash’ components and scene elements shared the same frequency pool.

The results of Experiment 7 are presented in Figure 10. The hit-rate and *d′* data both demonstrate that ‘splashes’ disrupted performance in both CA and CD stimuli. A repeated measures ANOVA with interruption type (non-interrupted vs. ‘splash’), change type, and scene size as factors revealed main effects of interruption (F(1,11) = 20.04, p = 0.001), change type (F(1,11) = 22.45, p = 0.001) and scene size (F(2,22) = 10.132, p = 0.001).

These effects cannot be attributed to adaptation because the ‘splashes’ were sufficiently spectrally distant (by at least 2 ERB) from the on-going scene components. Instead, the drop in performance is consistent with a role for sensitivity to transients in both appearance and disappearance processing - even a brief interference that draws attention away from the on-going scene is sufficient to disrupt change detection.

It is noteworthy that while gaps (Experiment 6, above) had not effect on CD performance, the introduction of ‘splashes’ (of the same duration) resulted in a substantial decline in disappearance detection. Both gaps and ‘splashes’ introduce competing transients that would mask the local transient specific to the scene change. It appears that the disruption by ‘splashes’ is more fatal than that due to insertion of gaps, potentially because ‘splashes’ constitute a new event that might be more effective at capturing attention away from the change-events. The RT data (below) are also consistent with this conclusion.

Response times (Figure 9C) for both CA and CD were significantly increased in the ‘splash’ condition. A repeated measure ANOVA on the RT data showed main effects of interruption (F(1,11) = 193.05, p<0.001), change type (F(1,11) = 21.63, p = 0.001) and scene size (F(2,22) = 40.46, p<0.001) as well as the following interactions: interruption×change type (F(1,11) = 10.66 p = 0.08), interruption×scene size (F(2,22) = 5.68, p = 0.01), change type×scene size (F(2,22) = 25.95, p<0.001) and interruption×change type×scene size (F(2,22) = 4.84 p = 0.018). While most of these effects are consistent with findings from the previous experiments, the interaction between interruption and change type is interesting because it indicates that CA RT performance was more affected than that for CD - Remarkably the introduction of the ‘splash’ nearly equated CA and CD response times. All other interruptions employed in this series of experiments (step changes in loudness, insertion of gaps; Experiment 3 and 6, respectively) still resulted in a sizeable CA RT advantage. The loss of CA response time superiority here indicates that the ‘splashes’ abolished appearance ‘pop out’.

## Discussion

Our results reveal a fundamental difference between ‘appear’ and ‘disappear’ events. Listeners are exceptionally sensitive to source appearance: change detection and identification are at ceiling, response times are short, with little effect of scene-size. In contrast, listeners have difficulty detecting, and still more so identifying, disappearing sources: Performance rapidly deteriorates with growing scene-size, response times are slow, and even when change is detected, the changed component is rarely successfully identified.

We introduced a variety of scene perturbations in order to understand the mechanisms supporting appearance and disappearance detection. Overall, our results demonstrate a role for neural adaptation and sensitivity to transients in the process of auditory change detection, similar to what has been demonstrated for visual change detection [1]–[6].

### Previous Work

Eramudugolla *et al* [11] used scenes of concurrently presented natural sounds instructing listeners to determine whether an item had disappeared following a 500 ms noise interruption. They demonstrated poor change detection, with performance decreasing with increasing scene-size. However performance improved significantly when listeners were primed ahead about the disappearing item. The authors conclude that listeners are ‘deaf to change’ and that top-down attention is essential for change detection in complex auditory scenes. Gregg and Samuel [9] studied item substitution. Changes involved the disappearance of an existing item and the appearance of a new one. However, contrary to our finding that listeners are highly tuned to appearing objects, they reported poor performance (error rate >40%). Similarly to the results presented here, Pavani and Turatto [10] found greater sensitivity to item addition than deletion for scenes of 4 items, but they attributed their results to limits of auditory short-term memory (detecting disappearance requires memorising 4 objects - one more than appearance) and argued that auditory change detection is fundamentally reliant on low-capacity memory resources. Their conclusion was also motivated by their finding that inserting silent- or noise-gaps at the time of change did not adversely affect performance, contrary to what would be expected for a low-level sensory constraint.

In contrast, our findings suggest that the previously reported ‘change deafness’ effects are specific to disappearance. The demonstration that CA performance is unaffected by increasing scene-size but (contrary to the findings in Pavani and Turatto [10]) perturbed by short scene interruptions suggests that appearance detection relies on low level, automatic, processes. The discrepancy with previous results is consistent with the possibility that those studies, by explicitly encouraging listeners to ‘label’ scene elements, at least partially tapped general working-memory limitations rather than intrinsic auditory change detection mechanisms (see also [27]).

Scene components in our stimuli were widely spaced in frequency so that inter-component energetic masking was minimal, and component addition/deletion was therefore accompanied by a clear increase or decrease in power in a particular frequency band. Minimizing energetic masking is essential because otherwise masking increases with scene size (and may thus result in reduced performance) – an effect which might have confounded previous results.

In addition to our use of acoustically simple stimuli, with no semantic attributes, the present design also differs from previous investigations [9]–[11] in that the signals were not spatialized. This simplifies the design and controls for spatial attention, so it is possible to concentrate on the spectral/temporal effects that contribute to change sensitivity.

The CA pop-out observed in our experiments is consistent with the ‘enhancement’ literature (e.g. [28]–[30]). In these experiments listeners are presented with complex tone stimuli, where one of the harmonics is alternately deleted (or reduced in amplitude) and then restored. This manipulation results in that harmonic being perceived as clearly popping out of the complex as an independent object against the background of the remaining, fused, harmonics. In a related experiment Bregman et al [31] (see also [32]) explored the perceptual salience of tone-onset in the context of random pure- tone chords and reported that when the onset of a pure-tone was desynchronized from the cluster, its pitch popped out of the otherwise fused mixture and dominated listeners' precepts. A similar, albeit weaker, effect was also observed for tone offset and interpreted as indicating that the auditory system is sensitive to local spectral transients introduced by sudden onsets or offsets. However, it has also been argued [e.g.10]. that sensitivity to local transients may not aid appearance or disappearance detection in natural acoustic environments because many sound sources are inherently characterized by energy fluctuations that would mask any genuine change-related transients. Our stimuli were specifically designed to model such fluctuating sources as a means for investigating change detection in realistically dynamic acoustic scenes. The present findings demonstrate that appearance pop out is a general phenomenon not restricted to the simple stimuli used previously.

### Detection of Item Appearance

The outstanding detection and identification performance, and its independence of growing scene-size, suggest an automatic process by which appearing components ‘pop-out’ and grab attention [33]. At least two kinds of low-level neural mechanisms may underlie these perceptual effects: (a) Local transients generated at signal onset (see ‘introduction to Experiment 6’) (b) adaptation effects - changes to the sustained neuronal firing rate [34]–[36] (see ‘introduction to Experiment 5’). Our results (Experiments 5, 6 and 7) provide evidence for both.

Notably, the prominence of ‘appear’ vs. ‘disappear’ events (as measured by hit rates, or *d′* scores) is maintained regardless of large-scale scene interruptions such as silent/noise gaps inserted at the time of change, prominent step-changes in overall loudness or acoustic ‘splashes’ (but see RT results in Experiment 7). The sustained CA advantage might be due to long-lasting effects of on-going adaptation which ‘survive’ the scene perturbation. However it could also be the case that CA dominance is enhanced at a later stage in the processing stream - for example, CA changes might possess an inherent perceptual advantage in attracting attention [33], [37], [38].

### Detection of Item Disappearance

Overall, listeners exhibited significant ‘disappearance blindness’ – missing about half of the changes in the larger scenes. In the absence of inter-component energetic masking as a confounding factor, the fact that performance on CD exhibited a strong dependence on scene size might suggest reliance on search, or memory - mechanisms which scan across the cell array for changes in activation (e.g. [13], [30], [33]). However data from the probe identification task (Experiment 4) suggest that the involvement of search in disappearance detection may be limited: Performance on CD identification was at floor - Even when change was detected, listeners were mostly unable to identify the changed component. This was the case even for the smallest scene size. A search based account would predict that identification performance should be equal to change detection performance. Similarly, the results of Experiment 5 demonstrate that disappearance detection (even in very small scenes) is not improved when listeners are given the opportunity to accumulate more information about scene contents (i.e. when changes occur late, rather than early, in the course of a scene), thus further rejecting search/memory based accounts.

However, the fact that CD performance was preserved following gap interruptions suggests that performance relies on some form of (albeit coarse) memory of the on-going scene. This is consistent with the subjective experience of listening to CD scenes. Disappearance detection seems to rely on non-specific cues (e.g. hearing a change in the general quality, or ‘timbre’, of the on-going scene signal) with no ‘feel’ for what exactly changed.

In contrast to gaps, the acoustic ‘splashes’ in Experiment 7 resulted in reduced CD performance. Because the ‘splashes’ were brief, and did not physically mask any of the scene components, it is unlikely that this drop in performance is caused by resetting of the memory representation discussed above. Instead, the data indicate that, despite the multitudes of onsets and offsets in our scene stimuli, disappearance detection is, similarly to appearance detection, partly based on sensitivity to change-related transients. Namely, there exists some form of temporary information, available briefly following the occurrence of the change, that when abolished (by the ‘splash’) results in a considerable decline (about 20%) in performance.

### Disappearance Blindness

Our experiments reveal a sizeable appearance (relative to disappearance) detection advantage which persists in the face of a variety of scene perturbations. Investigations in vision, have similarly reported a behavioral advantage for detecting appearing items, and argued that appearance might take attentional priority over disappearance [37] (but see [39]). This is also resonant of reports in audition, which reveal a perceptual bias for approaching (relative to receding) sounds [38]. However, as discussed above, even without invoking high level perceptual biases, there are likely to be lower level contributing factors to CA dominance. Detecting offset is a computationally harder problem: In our stimuli, component onsets are immediately apparent but to detect extrinsic offsets one must integrate for long enough so as to distinguish the intervals between pips from an actual signal cessation. Moreover, at the neural level, e.g. for a putative mechanism that scans the activation array for changes in firing rate, offsets (reduction in rate of a random process) are inherently harder to detect than onsets (increase in rate) [40]. These computational considerations, together with known properties of auditory neurons, such as adaptation or differences in onset-/offset- tuned cell populations might contribute to better performance on CA than CD. However, the notable aspect of the present results is the qualitative difference between appearance and disappearance detection, and the *degree* to which the auditory system is insensitive to item disappearance, even in very small scenes.

Detecting appearance is perhaps more immediately relevant for survival - it is certainly more important to detect a predator appearing in one's vicinity than that one just disappeared. However this reasoning does not apply to other, perhaps equally important situations (e.g. the sudden disappearance of the voice of your child in a crowd). Our data suggest that the auditory system does not possess mechanisms to efficiently deal with these situations, despite their apparent behavioural relevance. Rather than a ‘change detector’, the auditory system appears to be largely a novelty, or appearance detector.
